# A machine‐learned analysis of human gene polymorphisms modulating persisting pain points to major roles of neuroimmune processes

**DOI:** 10.1002/ejp.1270

**Published:** 2018-07-13

**Authors:** D. Kringel, C. Lippmann, M.J. Parnham, E. Kalso, A. Ultsch, J. Lötsch

**Affiliations:** ^1^ Institute of Clinical Pharmacology Goethe ‐ University Frankfurt am Main Germany; ^2^ Fraunhofer Institute for Molecular Biology and Applied Ecology IME Branch for Translational Medicine and Pharmacology TMP Frankfurt; ^3^ Institute of Clinical Medicine University of Helsinki Pain Clinic Helsinki University Central Hospital Helsinki Finland; ^4^ Institute of Biomedicine Pharmacology, University of Helsinki Helsinki Finland; ^5^ DataBionics Research Group University of Marburg Germany

## Abstract

**Background:**

Human genetic research has implicated functional variants of more than one hundred genes in the modulation of persisting pain. Artificial intelligence and machine‐learning techniques may combine this knowledge with results of genetic research gathered in any context, which permits the identification of the key biological processes involved in chronic sensitization to pain.

**Methods:**

Based on published evidence, a set of 110 genes carrying variants reported to be associated with modulation of the clinical phenotype of persisting pain in eight different clinical settings was submitted to unsupervised machine‐learning aimed at functional clustering. Subsequently, a mathematically supported subset of genes, comprising those most consistently involved in persisting pain, was analysed by means of computational functional genomics in the Gene Ontology knowledgebase.

**Results:**

Clustering of genes with evidence for a modulation of persisting pain elucidated a functionally heterogeneous set. The situation cleared when the focus was narrowed to a genetic modulation consistently observed throughout several clinical settings. On this basis, two groups of biological processes, the immune system and nitric oxide signalling, emerged as major players in sensitization to persisting pain, which is biologically highly plausible and in agreement with other lines of pain research.

**Conclusions:**

The present computational functional genomics‐based approach provided a computational systems‐biology perspective on chronic sensitization to pain. Human genetic control of persisting pain points to the immune system as a source of potential future targets for drugs directed against persisting pain. Contemporary machine‐learned methods provide innovative approaches to knowledge discovery from previous evidence.

**Significance:**

We show that knowledge discovery in genetic databases and contemporary machine‐learned techniques can identify relevant biological processes involved in Persitent pain.

## 1 Introduction

Persisting pain has a high prevalence (Elliott et al., 1999; Breivik et al., 2006; van Hecke et al., 2013) affecting a significant proportion of the world's population (Breivik et al., 2006). Its pathophysiology is incompletely understood, which is reflected in the limited success of available treatment options (Moore et al., 2009, 2012; Derry et al., 2012) and has stimulated intense research on this topic (Kringel and Lötsch, 2015). In this context, the study of human gene polymorphisms that modulate the persisting pain phenotype is an accepted research approach which has been pursued for more than 50 years (Godinova, 1965). A genetic background to persisting pain is clearly reflected by a protective effect against persisting pain exerted, for example, by a haplotype of the guanosine‐5′‐triphosphate (GTP) cyclohydrolase 1 gene (*GCH1*), originally reported to be composed of 15 genetic variants (Tegeder et al., 2006, 2008), or by a reduction in the perceived intensity of pain exerted, for example, by a deletion/insertion variant in the serotonin transporter gene‐linked polymorphic region (5‐HTTLPR; gene: *SLC6A4*) reportedly reducing the perception of heat pain (Lindstedt et al., 2011; Hooten et al., 2013; Kunz et al., 2016). On the other hand, increased risk for persisting pain is conferred, for example, by the rs12584920 variant of the 5‐hydroxytryptamine receptor 2A gene (*HTR2A*) (Nicholl et al., 2011) or the rs734784 polymorphism in the potassium voltage‐gated channel modifier, subfamily S member 1, gene (*KCNS1*) (Costigan et al., 2010).

Human genetic research during the last decade has identified many common variants of more than a hundred different genes spread across the genome that modulate the phenotype of persisting pain in several different clinical settings (Table 1). Thanks to concomitant developments in computer science, including progress in artificial intelligence, machine‐learning and knowledge discovery in databases (Ashburner et al., 2000), the analysis of fundamental, complex biological functions has become increasingly possible. This allows persisting pain to be approached at a functional genomics level by combining the information on genetic modulation acquired in clinical studies with current knowledge of the function of human genes. This active research topic has already led to the identification of candidate genes for further clinical genetic pain research (Lötsch et al., 2013) and highlighted key pathophysiological processes of pain which may be targeted for future pharmacological treatment options (Ultsch et al., 2016).

**Table 1:** List of 110 genes with empirically supported relevance to persisting pain, based on published evidence that their genetic variants are associated with phenotypic differences in persisting pain patients in several clinical settings

Gene	NCBI	Type of pain	References	Gene	NCBI	Type of pain	References	Gene	NCBI	Type of pain	References
*ACAN*	176	Musculoskeletal	Kirk et al. (2003)	*GCH1*	2643	Widespread	Kim et al. (2010a)	*MTHFD1*	4522	Musculoskeletal	Aneiros‐Guerrero et al. (2011)
*ACE*	1636	Musculoskeletal	Rommel et al. (2006)	*GDF5*	8200	Musculoskeletal	Valdes et al. (2011c)	*MTRR*	4552	Musculoskeletal	Aneiros‐Guerrero et al. (2011)
*ADRA1A*	148	Neuropathic	Herlyn et al. (2010)	*GNB3*	2784	Visceral	Oshima et al. (2010)	*MYT1L*	23,040	Widespread	Docampo et al. (2014)
*ADRA1D*	146	Visceral	Sugaya et al. (2002)	*GRK5*	2869	Miscellaneous	Smith et al. (2011)	*NCR3*	259,197	Neuropathic	Sato et al. (2002)
*ADRA2A*	150	Idiopathic	Kim et al. (2004)	*GSTM1*	2944	Visceral	Wu et al. (2000)	*NPY*	4852	Back pain	Herlyn et al. (2010)
*ADRA2C*	152	Idiopathic	Kim et al. (2004)	*GSTM1*	2944	Musculoskeletal	Aneiros‐Guerrero et al. (2011)	*NR3C1*	2908	Widespread	Holliday et al. (2010)
*ADRB2*	154	Widespread	Hocking et al. (2010)	*GSTP1*	2950	Visceral	Woo et al. (2010)	*NRXN3*	9369	Widespread	Docampo et al. (2014)
*ADRB2*	154	Musculoskeletal	Diatchenko et al. (2006)	*GSTT1*	2952	Visceral	Woo et al. (2010)	*NTRK1*	4914	Miscellaneous	Shatzky et al. (2000)
*ADRB2*	154	Neuropathic	Herlyn et al. (2010)	*HFE*	3077	Musculoskeletal	Alizadeh et al. (2007)	*OPRM1*	4988	Miscellaneous	Cheng et al. (2010)
*ADRB3*	155	Visceral	Sugaya et al. (2002)	*HLA‐A*	3105	Neuropathic	Sato et al. (2002)	*P2RX7*	5027	Musculoskeletal	Sorge et al. (2012)
*ANP32A*	8125	Musculoskeletal	Valdes et al. (2009)	*HLA‐B*	3106	Neuropathic	de Rooij et al. (2009)	*P2RX7*	5027	Neuropathic	Sorge et al. (2012)
*APOE*	348	Widespread	Reeser et al. (2011)	*HLA‐B*	3106	Inflammatory	Gullo et al. (1982)	*PCSK6*	5046	Musculoskeletal	Malfait et al. (2012)
*AR*	367	Visceral	Shaik et al. (2009)	*HLA‐C*	3107	Neuropathic	Ozawa et al. (1999)	*PGK1*	5230	Visceral	Riley and Krieger, 2002)
*ASPN*	54,829	Musculoskeletal	Nakamura et al. (2007)	*HLA‐DQA1*	3117	Neuropathic	de Rooij et al. (2009)	*PGR*	5241	Visceral	De Carvalho et al. (2007)
*CACNA2D3*	55,799	Back pain	Neely et al. (2010)	*HLA‐DQB1*	3119	Neuropathic	de Rooij et al. (2009)	*POMC*	5443	Widespread	Holliday et al. (2010)
*CACNG2*	10,369	Neuropathic	Nissenbaum et al. (2010)	*HLA‐DRB1*	3123	Neuropathic	Sato et al. (2002)	*PRSS1*	5644	Inflammatory	Midha et al. (2010)
*CALCA*	796	Neuropathic	Herlyn et al. (2010)	*HTR2A*	3356	Visceral	Pata et al. (2004)	*PTGS1*	5742	Visceral	Arisawa et al. (2008)
*CAMK4*	814	Miscellaneous	Smith et al. (2011)	*HTR3E*	285,242	Visceral	Kilpatrick et al. (2011)	*SCN5A*	6331	Visceral	Saito et al., 2009)
*CASP9*	842	Back pain	Guo et al. (2011)	*IFNG*	3458	Inflammatory	Noponen‐Hietala et al. (2005)	*SCN5A*	6331	Idiopathic	Reimann et al. (2010)
*CCT5*	22,948	Widespread	Peters et al. (2013)	*IFNG*	3458	Inflammatory	Oen et al. (2005)	*SCN9A*	6335	Back pain	Reimann et al. (2010)
*CFTR*	1080	Inflammatory	Midha et al. (2010)	*IFRD1*	3475	Miscellaneous	Smith et al. (2011)	*SCN9A*	6335	Inflammatory	Reimann et al. (2010)
*CRHBP*	1393	Widespread	Holliday et al. (2010)	*IL‐10*	3586	Back pain	Shoskes et al. (2002)	*SCN9A*	6335	Miscellaneous	Reimann et al. (2010)
*CILP*	8483	Back pain	Seki et al. (2005)	*IL‐10*	3586	Inflammatory	Noponen‐Hietala et al. (2005)	*SCN9A*	6335	Musculoskeletal	Valdes et al. (2011a)
*CNR1*	1268	Visceral	Park et al. (2011)	*IL‐10*	3586	Musculoskeletal	Oen et al. (2005)	*SCN10A*	6336	Neuropathic	Faber et al. (2012)
*COL1A1*	1277	Back pain	Tilkeridis et al. (2005)	*IL‐16*	3603	Visceral	Gan et al. (2010)	*SCN11A*	11,280	Widespread	Leipold et al., 2013)
*COL6A4P1*	344,875	Musculoskeletal	Miyamoto et al. (2008)	*IL‐1A*	3552	Back pain	Solovieva et al. (2004)	*SERPINA1*	5265	Widespread	Blanco et al. (2006)
*COL9A2*	1298	Back pain	Ala‐Kokko, 2002)	*IL‐1B*	3553	Back pain	Zhang et al. (2002)	*SERPINA6*	866	Widespread	Holliday et al. (2010)
*COL9A3*	1299	Back pain	Kales et al. (2004)	*IL‐1B*	3553	Miscellaneous	Jeremias et al. (2000)	*SHMT1*	6470	Idiopathic	Aneiros‐Guerrero et al. (2011)
*COMT*	1312	Musculoskeletal	van Meurs et al. (2009)	*IL‐1R2*	7850	Neuropathic	Stephens et al. (2014)	*SLC6A4*	6532	Idiopathic	Herken et al. (2001)
*COMT*	1312	Widespread	Cohen et al. (2009)	*IL‐1RN*	3557	Back pain	Kim et al. (2010b)	*SMAD3*	4088	Musculoskeletal	Valdes et al. (2010)
*COMT*	1312	Back pain	Dai et al. (2010)	*IL‐1RN*	3557	Musculoskeletal	Attur et al. (2010)	*SOD2*	6648	Idiopathic	Arisan et al. (2006)
*COMT*	1312	Visceral	Karling et al. (2011)	*IL‐1RN*	3557	Idiopathic	Witkin et al. (2002)	*SPINK1*	6690	Inflammatory	Midha et al. (2010)
*COMT*	1312	Idiopathic	Tahara et al. (2008)	*IL‐2*	3558	Inflammatory	Noponen‐Hietala et al. (2005)	*TAAR1*	134,864	Widespread	Smith et al. (2012)
*CRH*	1392	Widespread	Holliday et al. (2010)	*IL‐4*	3565	Visceral	Sugaya et al. (2002)	*TAC1*	6863	Back pain	Herlyn et al. (2010)
*CRHBP*	1393	Musculoskeletal	Linnstaedt et al. (2016)	*IL‐4*	3565	Inflammatory	Noponen‐Hietala et al. (2005)	*TACR1*	6869	Visceral	Renner et al. (2009)
*CRHR1*	1394	Widespread	Holliday et al. (2010)	*IL‐4R*	3566	Visceral	Sugaya et al. (2002)	*TGFB1*	7040	Back pain	Herlyn et al. (2010)
*CYP2D6*	1565	Visceral	Wu et al. (2000)	*IL‐4R*	3566	Inflammatory	Noponen‐Hietala et al. (2005)	*TGFB1*	7040	Inflammatory	Shoskes et al. (2002)
*DIO2*	1734	Musculoskeletal	Meulenbelt et al. (2008)	*IL‐6*	3569	Back pain	Herlyn et al. (2010)	*TGFB1*	7040	Musculoskeletal	Oen et al. (2005)
*DRD4*	1815	Musculoskeletal	Aneiros‐Guerrero et al. (2011)	*IL‐6*	3569	Idiopathic	Shoskes et al. (2002)	*TNF*	7124	Back pain	Herlyn et al. (2010)
*DRD4*	1815	Widespread	Buskila et al. (2004)	*IL‐6*	3569	Inflammatory	Noponen‐Hietala et al. (2005)	*TNF*	7124	Idiopathic	Shoskes et al. (2002)
*LPAR1*	1902	Musculoskeletal	Mototani et al. (2008)	*IL‐6*	3569	Neuropathic	Oen et al. (2005)	*TNF*	7124	Inflammatory	Noponen‐Hietala et al. (2005)
*ESR1*	2099	Idiopathic	Ribeiro‐Dasilva et al. (2009)	*KCNJ6*	3763	Back pain	Bruehl et al. (2013)	*TNF*	7124	Musculoskeletal	Oen et al. (2005)
*ESR1*	2099	Visceral	Govindan et al. (2009)	*KCNS1*	3787	Neuropathic	Costigan et al. (2010)	*TP53*	7157	Visceral	Ribeiro Junior et al. (2009)
*ESR1*	2099	Musculoskeletal	Kang et al. (2007)	*MAOA*	4128	Musculoskeletal	Gursoy et al. (2008)	*TPH2*	121,278	Widespread	Nicholl et al. (2011)
*FAM173B*	134,145	Widespread	Peters et al. (2013)	*MBL2*	4153	Idiopathic	Babula et al. (2004)	*TPH2*	121,278	Musculoskeletal	Nicholl et al. (2011)
*FKBP5*	2289	Musculoskeletal	Bortsov et al. (2013)	*MC1R*	4157	Idiopathic	Foster et al. (2004)	*TRPA1*	8989	Neuropathic	Binder et al. (2011)
*GBP1*	2633	Widespread	Smith et al. (2012)	*MC2R*	4158	Widespread	Holliday et al. (2010)	*TRPM8*	79,054	Neuropathic	Binder et al. (2011)
*GC*	2638	Visceral	Faserl et al. (2011)	*MIF*	4282	Visceral	Arisawa et al. (2007)	*TRPV1*	7442	Musculoskeletal	Valdes et al. (2011b)
*GCH1*	2643	Back pain	Doehring et al. (2009)	*SOD2*	6648	Visceral	Arisan et al. (2006)	*TRPV1*	7442	Neuropathic	Binder et al. (2011)

The gene, NCBI number, clinical setting of persisting pain are shown together with a key reference in which this association was reported. The studies are given grouped for the relevant gene, however, in arbitrary order of clinical settings or publication year.

**Figure 1:**
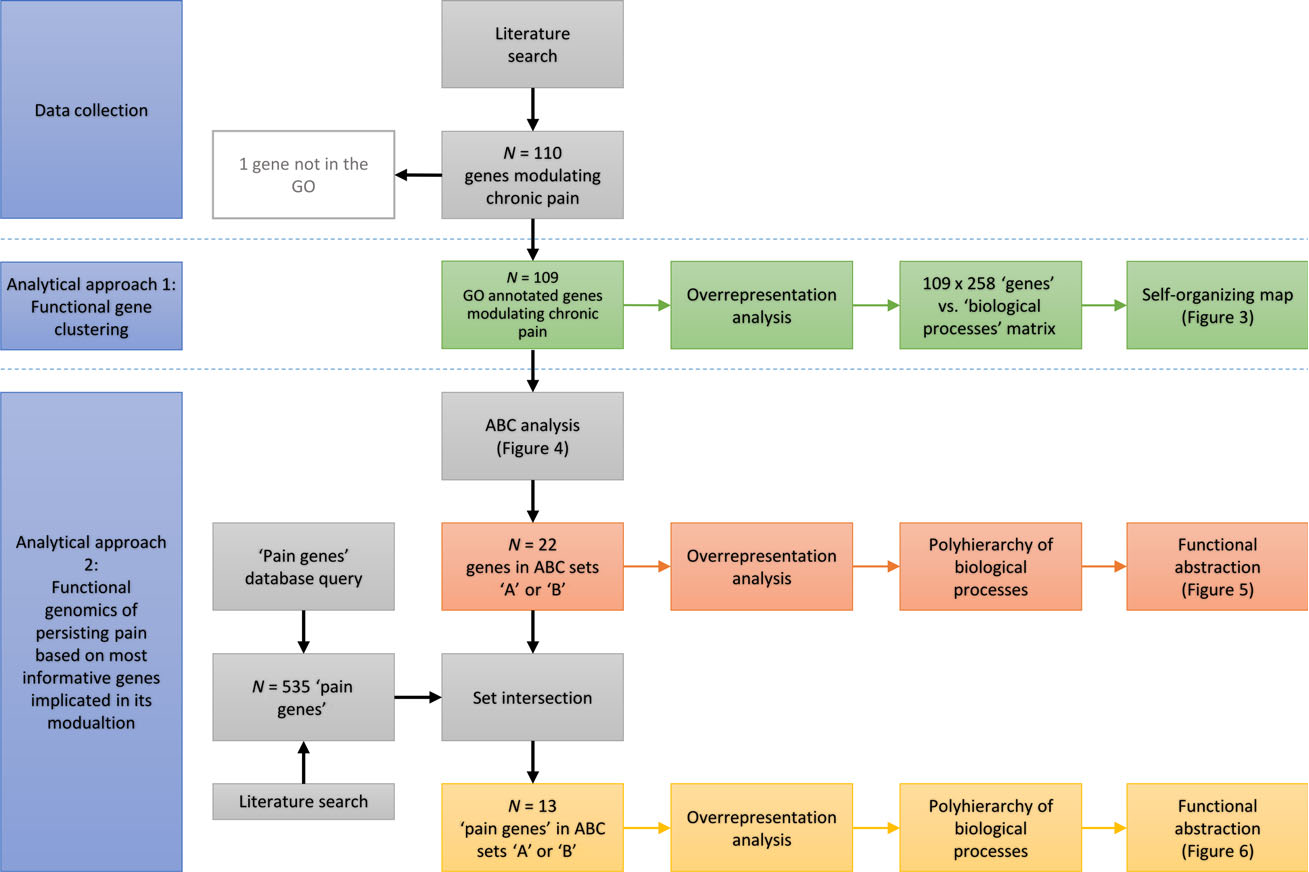
Flow chart of the data analysis. The figure provides an overview of the machine‐learning and data science approach applied, specifying the data flow of the gene sets and the analyses applied to them. The figure follows the analytical flow (left column of blue rectangles, separated by horizontal dashed lines) that following data collection from the literature was implemented as two analytical approaches comprising (1) functional gene clustering and (2) a computational functional genomics analysis of the biological roles of the genes implicated in the modulation of persisting pain in various clinical settings. The coloured frames show the presented analysis, the grey frames the intermediate data flows.

In the present analysis, information on genes, for which empirical evidence indicates the existence of variants that modulate the clinical phenotype of persisting pain, was analysed at a functional genomics level. In this way, the biochemical, cellular and/or physiological properties of each and every gene product can be investigated to gain an understanding of the relationship between the genome and the phenotype on a global genomewide scale (Gibson and Muse, 2009). Applying machine‐learned techniques (Fig. 1), the genes currently known to have relevance to human persisting pain were analysed for functional patterns that may provide insight into its pathophysiology based on current research activities, applying a data‐driven approach without using prior hypotheses about the most important biological functions characterizing persisting pain. By applying methods of precisely calculated item selection (Ultsch and Lötsch, 2015), the present analysis aimed to identify a subset of genes that were most consistently reported to be involved in the modulation of persisting pain with a subsequent analysis of the main biological functions exerted by the products of these genes.

## 2 Methods

### 2.1 Search strategy

A set of genes relevant to persisting pain, based on published associations of their variants with phenotypic differences in persisting pain patients, was obtained from (1) a PubMed database (accessed in August 2016), by searches for the string ‘(chronic OR persisting OR neuropathic OR back OR inflammatory OR musculoskeletal OR visceral OR widespread OR idiopathic OR fibromyalgia) AND pain AND (polymorphism OR variant) NOT review’ and (2) publications starting from the year 2000, which is close to the first sequencing of the human genome (Lander et al., 2001; Venter et al., 2001) marking the beginning of a new area of genetic research, and (3) published overviews on pain genetics (e.g. Edwards, 2006; Tegeder and Lötsch, 2009; Mogil, 2012; Zorina‐Lichtenwalter et al., 2016). To avoid redundancies, reports of positive associations of any gene variant were included only once per clinical setting in the present analysis. This implies that not every variant found to be functionally associated with a persisting pain phenotype was taken into account. The resulting information for each gene, thus, comprised (1) a positive report of a gene modulation in persisting pain and (2) the clinical setting of this finding.

### 2.2 Data analysis

Data were analysed using the R software package (version 3.3.2 for Linux; http://CRAN.R-project.org/; R Development Core Team, 2008) on an Intel Xeon^®^ computer running on Ubuntu Linux 16.04.1 64‐bit. The analysis employed several methods of machine‐learning that, as described previously (Lötsch and Ultsch, 2017aa), may be referred to as a set of methods that can automatically detect patterns in data and then use the uncovered patterns to predict or classify future data, to observe structures such as subgroups in the data or to extract information from the data suitable to derive new knowledge (Murphy, 2012; Dhar, 2013). More detailed descriptions including definitions of key concepts have been provided elsewhere (Lötsch and Ultsch, 2017aa).

The analysis aimed at describing the functional genomics of persisting pain based on the biological roles of the genes that reportedly carry variants modulating that phenotype. The biological roles were assessed as biological processes in which the genes are involved, defined as series of events or molecular functions with a defined beginning and end (Ashburner et al., 2000) and queried from the Gene Ontology (GO) knowledgebase that provides the acquired knowledge about the biological functions of gene products, described with a controlled vocabulary of GO terms (Ashburner et al., 2000).

Thus, the basis on which the present functional picture of persisting pain was created consisted of the biological processes in which the genes carrying modulatory variants were reported to be involved. The functional picture of persisting pain was sought pursuing two different analytical paths (Fig. 1). In a *first* approach, functional subgroups were sought in the set of human genes, variants of which have been associated with modulation of the clinical phenotype of persisting pain. This was addressed by applying a clustering algorithm on the matrix given by the genes versus their annotated biological processes; an approach that previously proofed as suitable for gene function based classifications (Lötsch and Ultsch, 2016a). In a *second* approach, the hypothesis was pursued that the functional genomics of persisting pain will prevail across clinical settings irrespective of the disease that had originally triggered the process. Therefore, the most informative subsets of the genes were identified using a computed item categorization technique (Ultsch and Lötsch, 2015) and subsequently, techniques of knowledge discovery were applied to identify the particular biological roles exerted by this set of genes as opposed to the biological functions exerted by a similarly sized random set of genes. This analysis was performed twice, once using the most informative genes resulting from above‐mentioned analysis, and, to strengthen the evidence for pain reliance, again using the additional selection criterion that the genes should be listed among pain‐relevant genes in the PainGenes database (Lacroix‐Fralish et al., 2007). The analytical steps are described in detail in the following.

#### 2.2.1 Functional clustering of genes

A first analytical approach to the functional genomics of persisting pain reflected in the set of genes that carry variants reported to modulate the phenotype aimed at finding functional clusters of genes.

#### 2.2.2 Generation of a gene versus functional feature matrix

As a prerequisite for functional clustering of genes, a gene versus function matrix was created. Therefore, the biological functions in which the genes, or their products, are involved were queried from the GO knowledge base (http://www.geneontology.org/). The GO knowledge base is searchable for three major categories, consisting of biological process, cellular component and molecular function. As the most suitable representation of processes that are involved in the chronification of central sensitization to pain, the GO category biological process was selected as previously (Lötsch et al., 2013; Lötsch and Ultsch, 2016b; Ultsch et al., 2016). According to the GO knowledgebase, this category contains one or more ordered collections of molecular functions involving chemical or physical transformations such as cell growth and maintenance or signal transduction (Ashburner et al., 2000).

**Figure 2:**
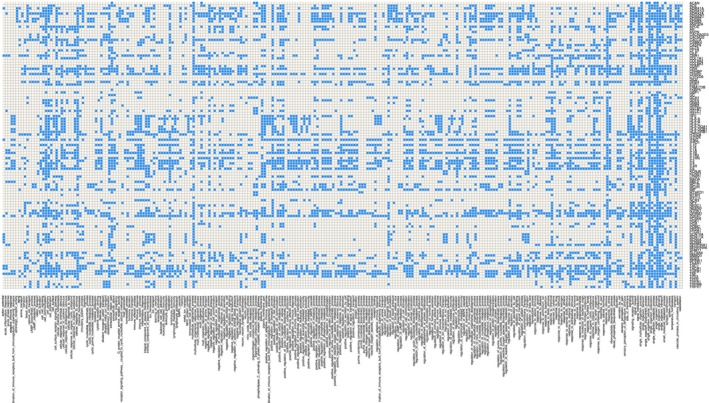
Matrix plot of the association of identified genes (rows; Table 1) with biological processes (columns), according to the annotations of the Gene Ontology (GO) knowledgebase (Ashburner et al., 2000), filtered for statistical significance in the present context by means of an overrepresentation analysis with a *p*‐value threshold of 1 × 10^−6^ and correction for multiple testing according to Bonferroni. The matrix displays a yes/no scaling [1,0], colour‐coded as blue or white, respectively. The figure has been created using the R software package (version 3.3.2 for Linux; http://CRAN.R-project.org/; R Development Core Team, 2008). Specifically, this plot was created using the ‘heatmap.2’ functions of the R package ‘gplots’ (Warnes G. R.; https://cran.r-project.org/package=gplots) with the build‐in clustering of the plotting routine disabled (R switches ‘Colv = FALSE, Rowv = FALSE’). For descriptions of the GO terms (abscissa), see the AmiGO search tool at http://amigo.geneontology.org/ (Carbon et al., 2009).

However, not all processes known to be influenced by the genes were sought, but only those that were annotated to the present set of genes more often than expected for any similarly sized random set of genes. Therefore, to capture biological processes that are particularly relevant to the present gene set, the set of genes was submitted to overrepresentation analysis (ORA; Backes et al., 2007). As intended, this compared the occurrence (as defined by its annotation term) of the particular set of genes covered by a GO term with the number of genes expected to be defined by this term. The significance of the association of a GO term with the expected list of genes was determined by means of a Fisher's exact test (Fisher, 1922). A *p*‐value threshold, *t*_*p*_, of 1 × 10^−6^ was applied and subsequently, the obtained results were additionally corrected for multiple testing according to Bonferroni (Hochberg, 1988). The result was the desired ‘gene versus biological process’ matrix (Fig. 2) that, rescaled as [0,1] indicating the absence or presence, respectively, of the involvement of a gene in a particular biological process, provided a filtered representation of the particularly important processes in which the analysed genes were involved while disregarding processes that would have been found by chance in any similarly sized gene set. This ORA‐based filtering of gene functions was previously found to facilitate the functional analysis of gene sets including a context of pain and analgesia (Lötsch and Ultsch, 2016a; Lötsch et al., 2017).

#### 2.2.3 Machine‐learned cluster detection

Following the creation of the gene versus functional feature matrix, expressed as ‘gene versus biological process’ matrix, the feature space required for functional gene clustering was established as $D=\{\left(x_{i}\right)|x_{i}\in N^{d},i=1,\ldots ,n\}$ comprising the *d* biological process to which the *n* genes in the analysed set were annotated. This feature space was searched for a cluster structure. Among several methods available for clustering, a method of unsupervised machine‐learning shown recently to provide a viable unbiased clustering of high‐dimensional biomedical data, outperforming classical clustering algorithms (Ultsch and Lötsch, 2017), was chosen. Specifically, the data space was projected from the high‐dimensional feature space *D* onto a two‐dimensional self‐organizing map (SOM) of the Kohonen type (Kohonen, 1982). This map was composed of a toroid grid (Ultsch, 2003), i.e. a projection plane where opposite edges are connected. The grid had a size of 25 × 35 artificial neurons chosen according to the proposals of SOM size determination described previously (Ultsch and Lötsch, 2017). Each of the artificial neurons held a position vector, which carried the information about the biological processes associated with each gene, and a further parameter, which carried ‘weights’ of the same dimensions as the input dimensions. The weights were initially randomly drawn from the range of the data variables and subsequently adapted to the data during the learning phase of 25 epochs. The Euclidean distance was used for process (dis‐)similarity; very general processes, such as ‘biological process’ that is the root term of the polyhierarchy carry the same value for all genes and therefore do not influence this distance. Following training of the neural network, an emergent SOM (ESOM; Lötsch and Ultsch, 2014; Ultsch and Sieman, 1990) was obtained that represented the genes as the localizations of their ‘best matching units’ (BMU), which are neurons that carried the vector most similar to a gene's data vector.

Following the projection of the data on the grid of neurons, an extension of the Kohonen map was applied to obtain clusters of genes. Specifically, the distance structure in the high‐dimensional space was visualized using the so‐called U‐matrix (Ultsch and Sieman, 1990; Lötsch and Ultsch, 2014). The clusters became visible using a geographical map analogy where ‘mountain ranges’ represent large distances in the feature space that can be used to visually separate data clusters, whereas low ‘valleys’ represent sets of genes that are related to similar biological processes and therefore belong to the same cluster. The ‘map’ was further enhanced by calculating a so‐called P‐matrix (Ultsch, 2003), which displays the probability of a data point as $p\left(x\right)=|\{\mathrm{data}\;\mathrm{points}\;x_{i}|d\left(x_{i},x\right)<=r\}|$ estimated as the number of data points in a sphere with radius *r* around *x* at each grid point on the ESOM's output grid. The calculations were performed using our R library ‘Umatrix’ (https://cran.r-project.org/package=Umatrix; Lötsch et al., 2018a).

#### 2.2.4 Functional genomics analysis of most informative genes reported to modulate persisting pain

A second analytical approach at the functional genomics of persisting pain reflected in the set of genes that carry variants reported to modulate the phenotype aimed at identifying the biological roles of those genes that had been implicated most consistently in this context. Specifically, the present analysis aimed at the discovery of new knowledge about pain, rather than about the underlying disease, from reports about a genetic modulation of a clinical symptom involving pain. This implies a distinction between the modulation of pain and the modulation of the disease‐causing pain that has usually not been made in the included reports. For example, in rheumatic diseases a genetic variant could modulate the progress and severity of inflammatory processes and via this it could finally modulate pain, or a genetic variant could directly modulate the individual perception of pain and therefore, similar nociceptive stimuli produced by the inflammatory processes could cause different degrees of pain. However, in the interest of the present analysis, we assessed only the direct modulations of pain, which were expected to be provided most likely by those genes that had been implicated in modulation of persisting pain in more than one clinical setting.

#### 2.2.5 Identification of genes most consistently reported to modulate persisting pain

To identify the most informative subset of genes reportedly modulating clinical persisting pain, a cut‐off criterion had to be defined for the number of different clinical settings required for inclusion in further analyses. To avoid arbitrary criteria, the cut‐off was obtained by applying an item categorization technique used to separate ‘the important few’ from the ‘trivial many’ (Juran, 1975). As a most suitable technique, because providing a mathematically based cut‐off, computed ABC analysis was chosen for the present purpose, supported by previous demonstrations that it is suitable for item selection tasks in biomedical research (Ultsch and Lötsch, 2015; Lötsch and Ultsch, 2017b; Lötsch et al., 2018b).

ABC analysis requires a set of positive numbers, which was given by the column sums of the ‘clinical settings versus genes’ matrix (Fig. 4 top). The vector of the sums of clinical settings with positive reports of the modulatory involvement a gene's variant was submitted to computed ABC analysis (Ultsch and Lötsch, 2015), which aims to divide a set of positive data – here the set of genes scored according to their involvement in clinical settings of persisting pain ‐ into three disjointed subsets called ‘A’, ‘B’ and ‘C’. Subset ‘A’ comprises ‘the important few’, subset ‘C’ comprises clearly nonprofitable values, i.e. ‘the trivial many’ (Juran, 1975), whereas subset ‘B’ includes items that provide still a balance between effort and gain. Therefore, the limit separating subset ‘C’, i.e. the genes for which a modulation of persisting clinical pain provides the least relevant information, was chosen as the limit for the inclusion of genes in further analyses. These calculations were made using our R package ‘ABCanalysis’ (https://cran.r-project.org/package=ABCanalysis; Ultsch and Lötsch, 2015).

#### 2.2.6 Functional genomics analysis of genes most consistently reported to modulate persisting pain

Following identification of the most informative genes as members of ABC sets ‘A’ or ‘B’, the functional genomics picture of persisting pain arising from these genes was analysed. This was obtained by applying ORA as described above, again using a *p*‐value threshold of *t*_*p*_ = 1 × 10^−6^ with Bonferroni α‐correction for multiple testing. The focus of this analysis was, however, the hierarchical representation of the complete knowledge on the biological roles of genes that carry polymorphisms observed to modulate persisting pain phenotypes. This was provided in a directed acyclic graph (DAG; Thulasiraman and Swamy, 1992). In this graph, the top‐down, branching polyhierarchy of GO terms starts with the most broadly defined terms and progresses towards the branches, representing GO terms with the narrowest definition (details). These calculations were made using our R package ‘dbtORA’ (https://github.com/IME-TMP-FFM/dbtORA; Lippmann et al., 2018), which has been designed for knowledge discovery in the GO.

As the complete DAG usually contains many GO terms (e.g. 64 GO terms in the present ORA), the information was transformed into a more intelligible form using the method of ‘functional abstraction’ (Ultsch and Lötsch, 2014). This aims to reduce the numbers of GO terms using a heuristic search algorithm that identifies so‐called functional areas (Ultsch and Lötsch, 2014), which are GO terms that qualify by their informational importance as headlines representing specific aspects (taxonomies) of the complete DAG with maximal coverage, precision, informational value and conciseness (Ultsch and Lötsch, 2014).

To narrow the focus even more to pain‐relevant genes, the ORA was repeated using the set intersection of the most informative genes identified as described above by means of ABC analysis, with the genes listed among known pain‐relevant genes in the PainGenes database (http://www.jbldesign.com/jmogil/enter.html; Lacroix‐Fralish et al., 2007). These mainly include genes found, in at least three independent studies in transgenic mice, to contribute to the modulation of pain and identified using PubMed searches, with the addition of further genes (Lötsch et al., 2013) comprising those causally implicated in human hereditary diseases associated with extreme pain phenotypes (summarized in, e.g. Lötsch et al., 2017), and genes coding for the targets of approved analgesic drugs or of novel analgesics currently in clinical phases of development (Lötsch and Geisslinger, 2011). This provided a set of *n* = 535 ‘pain genes’ (Fig. 1 bottom).

## 3 Results

As a result of a literature search, a total of 110 unique genes were identified in eight different clinical settings of chronic central sensitization to pain, including back pain, inflammatory pain, musculoskeletal pain, neuropathic pain, visceral pain, widespread pain, idiopathic pain and miscellaneous pain, for which functional associations of genetic variants with differences in the phenotype of persisting pain had been reported (Table 1). Some of the included studies used a genomewide approach; however, many were candidate gene studies.

### 3.1 Functional clustering of genes carrying variants reportedly modulating persisting clinical pain

In a first analytical approach, functional subgroups were sought in the set of human genes, variants of which have been associated with modulation of the clinical phenotype of persisting pain. A filtered representation of the particularly important processes in which the analysed genes were involved while disregarding processes that would have been found by chance in any similarly sized gene set, was obtained by means of overrepresentation analysis (ORA) of the biological processes to which the genes were annotated in the GO knowledgebase. This identified *d* = 258 biological processes (GO terms), given the *p*‐value threshold of 1 × 10^−6^ and the α‐correction according to Bonferroni. One gene was neglected in this analysis, *COL6A4P1*, the collagen type VI alpha 4 pseudogene 1, because it was not referenced in the GO.

**Figure 3:**
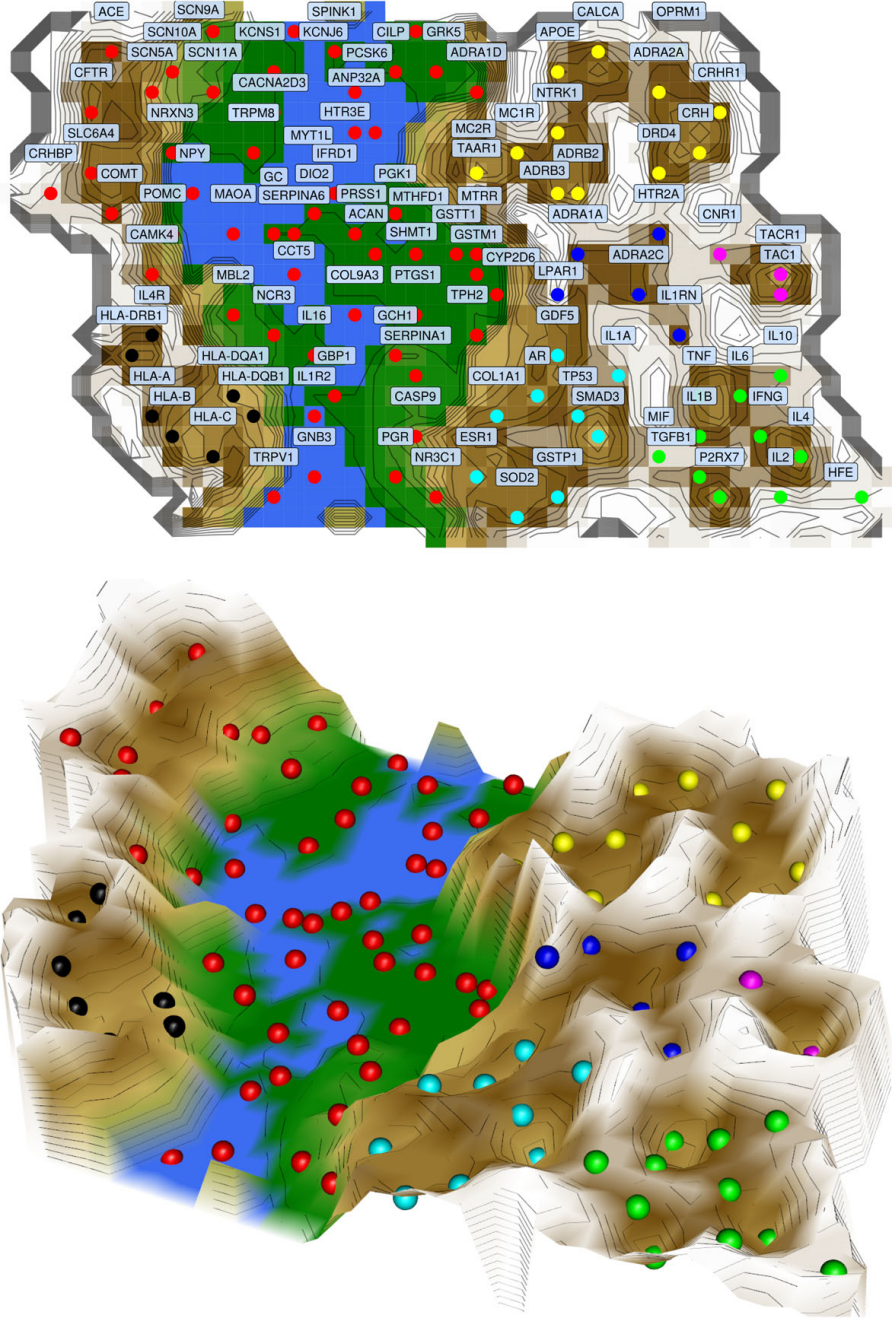
Result of a projection of the genes carrying variants reportedly modulating persistent pain onto a self‐organizing map (SOM) of the Kohonen type (Kohonen, 1982). Following projection of the genes on the grid of neurons, based on their functional annotations in the Gene Ontology knowledgebase (Ashburner et al., 2000) (*n* = 109; one of the 110 genes in Table 1 was not annotated in the GO), the distance and density structures in the high‐dimensional space were visualized using the so‐called U*‐matrix (Ultsch, 2003). Specifically, a trained SOM represents a topology preserving mapping of *n* high‐dimensional data points *x*_*i*_ *ε D*, where *D* denotes the data space, onto a two‐dimensional grid of neurons. A neuron *n* and the neurons in its neighbourhood *N(n)* on the output grid of the SOM represent points in the data space. The sum of distances between *n* and *N(n)* in the high‐dimensional space, combined with the respective density probabilities, is shown on a U*‐matrix as a height value (U‐height) at neuron *n*. Large U‐heights mean that there is a large gap in the data space. Low U‐heights mean that the points are close to each other within the data space (Lötsch and Ultsch, 2014). The dots indicate the so‐called best matching units (BMUs) of the SOM, which are those neurons whose weight vector is most similar to the input, i.e. the representation of the vector of the annotation of genes to GO terms. The BMUs are coloured according to the obtained clustering of the data space and labelled with the respective gene symbols. The cluster structure emerged from visualization of distances and density structures between neurons in the high‐dimensional space by means of a U*‐Matrix (Izenmann, 2009). Top: here, the genes represented by the BMUs are annotated. Bottom: 3D‐display of the U‐matrix in which the ‘valleys’, ‘ridges’ and ‘basins’ can be seen. Valleys indicate clusters of functionally similar genes based on the significant GO term annotations. The figure was created using the R software package (version 3.3.2 for Linux; http://CRAN.R-project.org/; R Development Core Team, 2008) using our R library ‘Umatrix’ (https://cran.r-project.org/package=Umatrix; Lötsch et al., 2018a).

Subsequently, the 109 × 258 sized ‘gene to biological process matrix’ (Fig. 2) thus obtained was analysed for functional subgroups of genes. Using unsupervised machine‐learning implemented as self‐organizing artificial neuronal network of the Kohonen type enhanced by the U‐matrix (i.e. an emergent self‐organizing map, ESOM; Ultsch and Sieman, 1990; Lötsch and Ultsch, 2014), the high‐dimensional data space was projected onto a two‐dimensional toroid grid. On this grid, the U‐matrix was visualized by applying a geographical landscape analogy (Fig. 3) providing a visual structure that could be employed for clustering of genes. The results indicated a large cluster comprising more than half of the genes (*n* = 58). However, this cluster was still composed of functionally very different genes, pointing towards a large heterogeneity of the genes chosen as candidate modulators of persisting pain in the different clinical studies, or was found with genomewide association studies (GWAS) without a focus on a particular gene. In addition, six smaller clusters were suggested, but their distinct separation was occasionally incomplete or, according to the U‐matrix landscape analogy, they were not clearly presented as ‘valleys’ but consisted merely of ‘mountain’ zones separated by slightly higher ridges (Fig. 3). In ESOM/U‐matrix based clustering, this indicates rather large intracluster distances.

As the present method has been shown to be well able to identify existent cluster structures while being unlikely to show false clusters (Ultsch, 2005; Ultsch and Lötsch, 2017), the main result of this analysis was that there is considerable heterogeneity among the genes reported to be involved in persisting pain without a clear functional focus on common general processes underlying this trait.

### 3.2 Functional genomics analysis of most informative genes reported to modulate persisting pain

In a second analytical approach, the hypothesis was pursued that the functional genomics of persisting pain will prevail across clinical settings irrespective of the disease that had originally triggered the process. However, the set intersection of the genes associated with any of the eight different clinical settings was empty. Nevertheless, the hypothesis that modulatory effects on chronic central sensitization to pain, rather than on the underlying disease‐causing pain, are exerted by the same genes across several clinical settings could be pursued in several genes have been shown to be involved in more than one clinical setting of chronic central sensitization.

**Figure 4:**
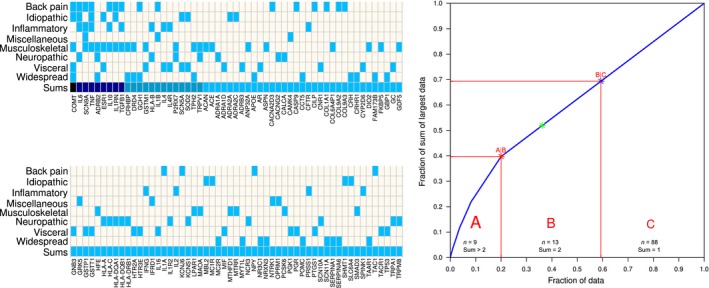
Number of clinical settings in which variants in the respective genes have been reported to be associated with modified phenotypes of chronic pain. Left: Matrix plot of the clinical settings (rows) versus the genes (*n* = 110, columns split in two halves, sorted for column sums in descending order). The column sums are displayed in the bottom row below the matrix. The numbers are displayed colour‐coded with 0 = white, 1 = blue, >1 darker blue). Right: ABC plot of the cumulative distribution function of the sums of clinical settings in which the genes were reportedly involved (column sums in the top matrix; bottom line). The ABC set limits are indicated as red lines (for further details about an ABC analysis, see (Ultsch and Lötsch, 2015)). The figure was created using the R software package (version 3.3.2 for Linux; http://CRAN.R-project.org/; R Development Core Team, 2008). Specifically, the matrix plot was created using the ‘heatmap.2’ functions of the R package ‘gplots’ (Warnes G. R.; https://cran.r-project.org/package=gplots), with the build‐in clustering of the plotting routine disabled (R switches ‘Colv=FALSE, Rowv=FALSE’). The ABC curve was drawn using our R library ‘ABCanalysis’ (https://cran.r-project.org/package=ABCanalysis; Ultsch and Lötsch, 2015).

To identify how many clinical settings qualify as a cut‐off, an ABC analysis was applied to the sum of settings in which each of the 110 genes was involved (column sums in the matrix plot in Fig. 4, top). This assigned 22 genes to ABC sets ‘A’ or ‘B’ (Table 2) that were included in further analysis as they could be regarded as best suited to perform an evidence‐based functional genomics analysis of chronic central sensitization to pain, considering the difficulty of separation between a modulation of the pain‐causing disease from a modulation of the perception and processing of chronic nociceptive input when a gene was involved only in a single clinical setting of persisting pain.

**Table 2:** Genes for which functional involvement of their variants has been reported in more than one clinical setting of persisting pain (number of these clinical settings given in the third column) were assigned to ABC sets ‘A’ or ‘B’ (Figure 4), comprising the most profitable information for an evidence‐based functional genomics analysis of persisting pain

Gene symbol	Gene name	Number of clinical settings of persisting pain	Functional areas						
			Response to stimulus	Immune system process	Reactive oxygen species metabolic process	Transport	Neurological system process	Regulation of multicellular organismal process	Multiorganism process
*COMT*	Catechol‐O‐methyltransferase	5	X	O	O	X	X	X	X
*IL‐6*	Interleukin‐6	4	X	X	X	X	X	X	X
*SCN9A*	Sodium channel, voltage‐gated, type IX alpha subunit	4	X	O	O	X	X	O	O
*TNF*	Tumour necrosis factor	4	X	X	X	X	X	X	X
*ADRB2*	Adrenoceptor beta 2, surface	3	X	O	O	X	O	X	O
*ESR1*	Oestrogen receptor 1	3	X	O	X	O	O	X	X
*IL‐10*	Interleukin‐10	3	X	X	X	X	X	X	X
*IL‐1RN*	Interleukin‐1 receptor antagonist	3	X	X	O	X	X	X	X
*TGFB1*	Transforming growth factor, beta 1	3	X	X	X	X	O	X	X
*CRHBP*	Corticotropin‐releasing hormone binding protein	2	X	O	O	X	X	X	X
*DRD4*	Dopamine receptor D4	2	X	O	O	X	X	X	X
*GCH1*	GTP cyclohydrolase 1	2	X	X	X	O	X	O	X
*GSTM1*	Glutathione S‐transferase mu 1	2	X	O	O	O	O	O	O
*HLA‐B*	Major histocompatibility complex, class I, B	2	X	X	O	O	O	X	X
*IL‐1B*	Interleukin‐1, beta	2	X	X	X	X	O	X	X
*IL‐4*	Interleukin‐4	2	X	X	X	X	O	X	X
*IL‐4R*	Interleukin‐4 receptor	2	X	X	O	X	O	X	X
*P2RX7*	Purinergic receptor P2X, ligand gated ion channel, 7	2	X	X	X	X	X	X	X
*SCN5A*	Sodium channel, voltage‐gated, type V alpha subunit	2	X	O	O	X	X	X	O
*SOD2*	Superoxide dismutase 2, mitochondrial	2	X	X	X	O	X	O	X
*TPH2*	Tryptophan hydroxylase 2	2	X	O	O	O	O	O	O
*TRPV1*	Transient receptor potential cation channel, subfamily V, member 1	2	X	O	O	X	X	O	O
	Sum		22	12	10	16	13	16	16

**Figure 5:**
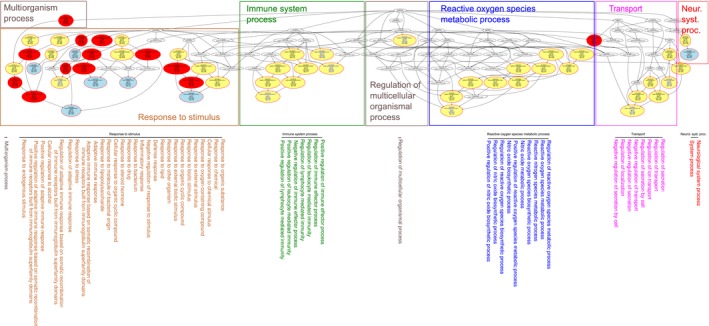
Results of an overrepresentation analysis (ORA;* p*‐value threshold, *t*_*p*_ = 1 × 10^−6^ and Bonferroni α correction) of 22 genes (Table 2) carrying variants reported to modulate the persisting pain phenotypes in different clinical settings (ABC sets ‘A’ or ‘B’) versus all human genes. A top‐down representation of the annotations (GO terms) is shown representing a systems‐biology perspective of the biological processes modulated by this set of genes. Each ellipse represents a GO term. The graphical representation follows the standard of the GO knowledge base, where GO terms are related to each other by ‘is‐a’, ‘part‐of’, and ‘regulates’ relationships forming a branching polyhierarchy organized in a directed acyclic graph (DAG; Thulasiraman and Swamy, 1992). Top: Significant GO terms are shown as coloured ellipses with the number of member genes, the number of expected genes by chance and the significance of the deviation in the observed from the expected number of genes indicated. The biological processes in which the present *n* = 22 genes are involved can be summarized by seven primary ‘functional areas’ or headlines presenting particular aspects (taxonomies) of the complete polyhierarchy at maximum coverage, precision, informational value and conciseness (Ultsch and Lötsch, 2014). The ellipses are colour‐coded using yellow for a ‘headline’; i.e. a GO term that by its location in the polyhierarchy may serve as headlines for a branch of the hierarchy, red for significantly overrepresented terms and white for nonsignificant terms that need to be displayed to preserve the polyhierarchical structure of the DAG. Blue vertices or blue labels are the most specific terms (leaves of the DAG) at the end of a taxonomy (branch) in the polyhierarchy. Bottom: The GO terms (biological processes) taken from the functional areas, shown above in the DAG, are shown with larger fonts for better readability. The figure was created using the R software package (version 3.3.2 for Linux; http://CRAN.R-project.org/; R Development Core Team, 2008) and our R package ‘dbtORA’ (https://github.com/IME-TMP-FFM/dbtORA; Lippmann et al., 2018).

The right part of the table displays the functional areas (Figure 5) or groups of biological functions in which the gene set is involved, together with their association with each gene (X = yes, O = No). The precise definition of the GO terms can be obtained using AmiGO search tool for GO at http://amigo.geneontology.org/ (Carbon et al., 2009).

ORA identified 64 GO terms associated with this particular subset of genes more often than expected by chance, given the chosen *p*‐value thresholds of 1 × 10^−6^ and correction for multiple testing according to Bonferroni. These terms provided a functional genomics perspective of the genes with variants shown to modulate the persisting pain sensitization phenotype in different clinical settings. Their graphical representation visualized their arrangement in the GO polyhierarchy (Fig. 5). Following application of functional abstraction (Ultsch and Lötsch, 2014), the main biological functions of the 22 gene carrying variants modulating persisting pain, were grouped around seven centres of biological activity or functional areas (Table 2; Fig. 5).

A first functional area to emerge was ‘immune system process’, represented in this particular gene set as an important common biological function (Fig. 5 middle left). The most significantly associated immune regulatory processes were ‘regulation of immune effector processes’ (GO:0002697, *p* = 2.8 × 10^−8^) and ‘positive regulation of lymphocyte mediated immunity’ (GO:0002708, *p* =  2.9 × 10^−7^). A second functional area centred on ‘reactive oxygen species metabolic process’ (GO:0072593, *p* = 4.5 × 10^−11^) and comprised mainly nitric oxide signalling related processes (Fig. 5 middle right) such as ‘nitric oxide biosynthetic process’ (GO:0006809, *p* = 3.2 × 10^−12^) and its regulation.

Further functional areas, however, mainly reflected processes known from previous research to contribute to pain (Lötsch et al., 2013; Ultsch et al., 2016). This included ‘response to stimulus’ with several subordinate terms, such as ‘response to stress’ (GO:0006950, *p* = 5.2 × 10^−7^), comprising the reaction of the body to several challenges such as ‘response to chemical’ (GO0042221, *p* = 1.1 × 10^−10^) and ‘defense response’ (GO:0006952, *p* = 1.8 × 10^−7^). Further subordinate areas included ‘inflammatory response’ (GO:0006954, *p* = 2.8 × 10^−8^) and ‘response to other organism’ (GO:0051707, *p* = 2.5 × 10^−10^) to which ‘response to bacterium’ (Fig. 5 middle left) was related. These response areas were also associated with the more general functional area ‘regulation of multicellular organismal process’ (GO: 51239, *p* = 1.2 × 10^−7^). In addition, the associated functional area ‘transport’, mainly comprised subordinate processes related to ion or transmitter transport such as ‘regulation of ion transport’ (GO:0043269, *p* = 9 × 10^−9^) or ‘regulation of secretion by cell’ (GO:1903530, *p* = 4.1 × 10^−7^). Finally, a functional area ‘neurological system process’ (GO:0050877, *p* = 2.4 × 10^−7^), as a more specific subordinate term to ‘system process’, reflected the expected involvement of nervous system related processes with pain.

**Figure 6:**
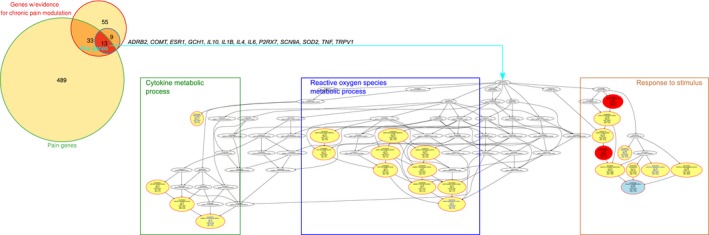
Overrepresentation analysis (ORA) of 13 genes (Table 2), versus all human genes, that (1) carry variants reported to modulate the persisting pain phenotypes, (2) belonging to the subset of 22 of these genes that are supported by evidence that this modulation applies to several clinical settings (ABC sets ‘A’ or ‘B’) and (3) were also part of a set of 535 genes, based on current pain research data, that can be considered, with their products, to be involved in the pathophysiology of pain (Ultsch et al., 2016). Left part: The Venn diagram (Venn, 1880) shows the set intersections on which this selection of 13 genes is based. Right part: Top‐down representation of the annotations (GO terms) representing the systems‐biology perspective of the biological processes modulated by this set of genes organized in a branching polyhierarchy forming a directed acyclic graph (DAG; Thulasiraman and Swamy, 1992). The figure represents the results of an overrepresentation analysis with parameters for *p*‐value threshold, *t*_*p*_ = 1 × 10^−6^ and Bonferroni α correction. The biological processes in which the *n* = 13 genes are involved can be summarized by three primary ‘functional areas’ or headlines presenting particular aspects (taxonomies) of the complete polyhierarchy at maximum coverage, precision, informational value and conciseness (Ultsch and Lötsch, 2014). The ellipses are colour‐coded using yellow for a ‘headline’; i.e. a GO term that by its location in the polyhierarchy may serve as headlines for a branch of the hierarchy, red for significantly overrepresented terms located in the polyhierarchy below a headline and white for nonsignificant terms that need to be displayed to preserve the polyhierarchical structure of the DAG. Blue vertices or blue labels are the most specific terms (leaves of the DAG) at the end of a taxonomy (branch) in the polyhierarchy. Bottom: The GO terms (biological processes) taken from the functional areas, shown above in the DAG, are shown with larger fonts for better readability. The figure was created using the R software package (version 3.3.2 for Linux; http://CRAN.R-project.org/; R Development Core Team, 2008) and our R package ‘dbtORA’ (https://github.com/IME-TMP-FFM/dbtORA; Lippmann et al., 2018). The Venn diagram was drawn using the R library ‘Vennerable’ (Swinton J., https://r-forge.r-project.org/R/?group_id=474).

The main results, i.e. the most specific functional areas pointing at immune processes and nitric oxide signalling as key biological processes involved in persisting pain across more than a single clinical setting, prevailed when narrowing the gene set further on those that are also listed in the PainGenes database (Fig. 6). As the set intersection between the 22 genes identified above and the genes of the PainGenes database included only *n* = 13 genes, the ORA applying the same statistical thresholds resulted in fewer additional significant GO terms (Fig. 6).

## 4 Discussion

The present analysis used empirical evidence for functional human genetic variants to approach the genetic architecture of persisting pain. Although the evidence was collected from separate studies, its combination permitted a limited genomewide association analysis of the trait. Methods for data mining and machine‐learned knowledge discovery were applied to publicly available databases in order to relate knowledge, acquired in the context of clinical pain research, on genes that modulate the phenotype of persisting pain with data on the biological functions of these human genes acquired in any context, without restriction to pain research (Lötsch et al., 2013).

The initial analysis of the whole set of genes showing positive results from clinical pain association studies indicated that, apart from a minority of genes that could be topically grouped such as interleukin or histocompatibility complex‐related genes, most genes displayed very heterogeneous functions and the analysis did not illuminate the pathophysiology of persisting pain beyond the functions of the single genes. This was probably due to the fact that data were drawn mainly from candidate gene or GWAS approaches. This selection probably introduced a research bias by (1) addressing genetic modulators in the context of the underlying disease and (2) including a selection of genetic markers that mimic other successful reports of comparable studies.

The situation became clearer when the analytical focus was narrowed to genetic modulations consistently observed across several clinical conditions with potential underlying painful diseases. This reduced the analytical bias generated by genetic modulations responsible for a specific pain‐causing disease and is in keeping with the contemporary approach to persisting pain as a distinct condition of central sensitization to pain and not merely a symptom of another underlying chronic disease. Consequently, it would be expected that the trait is modulated by specific genes which should be reflected by observations on its modulation in clinical research. The mathematically precise calculation provided by the ABC analysis (Ultsch and Lötsch, 2015), developed in order to select the most promising or profitable items from a larger set of items, resulted in identification of a set of 22 genes which could be then be assessed in a computational functional genomics analysis of persisting pain.

A major finding of this analysis of available evidence on genetic modulation of persisting pain was the particular importance of two groups of biological processes indicating involvement (1) of the immune system and of (2) nitric oxide signalling in persisting pain. Involvement of both processes is biologically highly plausible; however, their emergence as major process groups from a functional genomics analysis of data from clinical genetic research on persisting pain was not anticipated. Specific roles for the present subset of 22 genes, with repeated evidence for involvement in persisting pain, were exhibited by the 12 genes annotated as ‘immune system process’ (Table 1). This subset included interleukin (*IL‐1B*,* IL‐4*,* IL‐6*,* IL‐10*) (Dinarello, 1994; Choi and Reiser, 1998; Mocellin et al., 2004; Nemeth et al., 2004), interleukin receptor (*IL‐1RN*,* IL‐4R*) (Bittar and Bittar, 1996) and histocompatibility complex‐related (*HLA‐B*) genes (Dupont and Ceppellini, 1989), which have been shown to be involved in immunological mechanisms of pain (Sato et al., 2002; de Rooij et al., 2009). This is also supported by published evidence for the further genes in this list, such as, *TNF* (Vassalli, 1992; Franchimont et al., 1999), *TGFB1* (Li et al., 2006), *GCH1* (Schott et al., 1993), *P2RX7* (Schwartz et al., 2009) and *SOD2* (Wells et al., 2003). The second major process group emerging from the functional genomics analysis of the key evidence for genetic modulation of clinical persisting pain was nitric oxide signalling, in particular metabolic processes, summarized in this context under the GO term ‘reactive oxygen species metabolic process’ which includes the genes *IL‐6* (Deakin et al., 1995), *TNF* (Deakin et al., 1995; Katusic et al., 1998), *ESR1* (Clapauch et al., 2014), *IL‐10* (Cattaruzza et al., 2003), *TGFB1* (Saura et al., 2005), *GCH1* (Katusic et al., 1998; Zhang et al., 2007), *IL‐1B* (Katusic et al., 1998), *IL‐4* (Coccia et al., 2000), *P2RX7* (Gendron et al., 2003), *SOD2* (Fridovich, 1978). It is widely accepted that nitric oxide (NO) is critically involved in persisting pain (Chung, 2004). It has been shown that NO is produced in the spinal dorsal horn neurons in response to extensive nociceptive inputs and then it diffuses out and increases neurotransmitter release from primary afferent terminals, thereby contributing to central sensitization and persisting pain (Lin et al., 1999). Recent findings seem to indicate that not only NO is a mediator of persisting pain that accompanies inflammation, other reactive oxygen species like superoxide (SO) might also participate in persisting pain (Schwartz et al., 2008). Kim and colleagues found that NO and SO contribute to persisting pain via two separate and independent pathways and a recent study has shown that capsaicin‐induced hyperalgesia is a consequence of superoxide build‐up in spinal dorsal horn neurons. Superoxide dismutase (SOD‐2) encoded by gene *SOD2* is a major determinant suggesting a therapeutic potential for the manipulation of spinal SOD‐2 activity in pain conditions (Schwartz et al., 2009).

The role of cytokines was further highlighted by further restricting the gene subset most consistently related to persisting pain by identifying the appearance of the relevant gene in an independently created list of 535 so‐called pain genes (Ultsch et al., 2016). These were genes relevant to pain listed by several sources: mainly the Pain Genes Database (http://www.jbldesign.com/jmogil/enter.html (Lacroix‐Fralish et al., 2007). The overlap of the set of the 22 genes, with repeated records of variants that modulate the clinical phenotype of persisting pain, with the 535 ‘pain genes’ comprised 13 genes (*ADRB2*,* COMT*,* ESR1*,* GCH1*,* IL‐10*,* IL‐1B*,* IL‐4*,* IL‐6*,* P2RX7*,* SCN9A*,* SOD2*,* TNF*,* TRPV1*). After performing a similar ORA (Fig. 6) as that described above, these 13 genes were found to be mainly involved in cytokine production, covered by the significant GO terms ‘chemokine metabolic process’ (GO:0050755, *p* = 3.3 × 10^−7^), ‘nitrogen compound metabolic process’ and again ‘response to stimulus’, the most significant term being ‘nitric oxide biosynthetic process’ (GO:0006809, *p* = 1 × 10^−14^).

The involvement of the immune system in persisting pain is plausible from a biological perspective. One of the sites of interaction of the immune system with persisting pain has been identified as neuroimmune crosstalk at the glial–opioid interface (Tian et al., 2012). Previous research has shown that glial and immune cells, including astrocytes, microglia/macrophages, as well as T lymphocytes, are key cells activated during persisting pain, which contribute to pain persistence (Calvo et al., 2012; von Hehn et al., 2012). A role for local production of cytokines in the central nervous system during inflammatory conditions associated with persisting pain, such as rheumatoid arthritis (Lampa et al., 2012) or fibromyalgia (Kadetoff et al., 2012), as well as evidence for central nervous system sensitization by cytokines (Aden et al., 2010) also suggests such an immune system interaction with persisting pain states. Indeed, it has recently been suggested that the predominance of pain sensitization during chronic diseases in women is closely linked to the effects of female sex hormones on the neuroimmune system (Rosen et al., 2017).

The present results clearly support the modulation of neuroimmune system processes as a promising strategy in the development of novel analgesic drugs against persisting pain. This may be possible along several lines. For example, involvement of glial cells in opiate actions has been shown recently (Chen et al., 2010; Boue et al., 2012). Consequently, the elucidation of pain‐ and opioid‐induced mechanisms at the level of glial and immune cells could lead to improvement of pain management. In an animal model, ibudilast, a nonselective phosphodiesterase‐inhibiting, anti‐inflammatory drug that also blocks glial activation probably via antagonism at the Toll‐like receptor 4 (Jia et al., 2012), restored morphine‐induced antinociception following tolerance development (Lilius et al., 2009). Similarly, minocycline, a tetracycline that inhibits microglial activation and proliferation, also seems to attenuate morphine tolerance in mouse models of neuropathic pain (Chen et al., 2010). Hence, increasing evidence points towards the immune system as a potential source of future targets for analgesic drugs directed against persisting pain.

By gathering the relevant reports, the present analysis centres on the current evidence about a genetic modulation of persisting pain on a gene level, without going into the details of single nucleotide polymorphism level as usually applied in review papers. However, the approach was centred on machine‐learned knowledge discovery from the gathered evidence and was based on published evidence gathered from studies in which the authors had used a candidate gene approach or had performed a GWAS without a gene‐specific hypothesis. Therefore, the present analysis implies a research bias given by the original hypotheses or on the inclusion of frequently addressed genetic variants in the analysed studies. The question addressed with the present analysis was about the greater functional perspective emerging from successful clinical studies of the genetic modulation of persisting pain. Importantly, while the analysis is biased with respect to the gene selection, made by the authors of the included studies, its results are not biased for a particular functional genomics perspective as this had not been an gene inclusion criterion in the analysed studies. Nevertheless, the present selection of repeatedly reported associations implies an advantage of frequently included genes that have been attracted research interest through the last several years, such as *OPRM1*,* GCH1* or *COMT*.

With the caution advised by the implicit research bias regarding the gene selection, the results of the present analysis were (1) unexpected considering that hypotheses about the involvement of immune system processes or of nitric oxide signalling were not preformulated for the present analysis and (2) biologically plausible and completely compatible with current research activities on persisting pain in the light of increasingly acknowledgement of an involvement of immune processes that has attracted concerted research activities (Kringel and Lötsch, 2015), including that on the role of the glial–opioid interface in persisting pain (http://gloria.helsinki.fi). Hence, although the evidence was generated in separate studies, the combination of positive findings of a genetic modulation of persisting pain allows a limited yet valid genomewide analysis of the trait, providing a more comprehensive picture of functional background of persisting pain from genomics perspective than associations of single genotypes.

## 5 Conclusions

While many studies have focused on particular genes, the present analysis pursued the question whether their combined results may provide more complex insights into the pathophysiology of persisting pain in humans. In keeping with the contemporary trend towards ‘big data’ analysis in biomedical research, the current empirical data on modulation of persisting pain via human genetic polymorphisms have been subjected here to a computational functional genomics analysis. This evidence was then analysed for emergent, principal pathophysiological processes that characterize persisting sensitization to pain. Analysis of 110 unique genes, with variants that have been reported to modulate the clinical phenotype of persisting pain, led to the selection of functionally heterogeneous genes. By focusing on genes that have been repeatedly associated with modulation of persisting pain phenotypes in several clinical settings, a clearer picture emerged of the main processes identified by current human genetics research on persisting pain. A mathematically supported, precise selection of a subset of genes was possible using a computational functional genomics approach. On this basis, including the research bias of current clinical genetic association studies, the evidence gathered so far points to the view of persisting pain as a trait resulting from alterations in the immune system and/or in nitric oxide signalling, a concept that is biologically highly plausible and agrees with other lines of pain research. While analysing existing evidence and therefore limited to previously shown functional pathways, the present computational functional genomics‐based approach provides a computational systems‐biology perspective on chronic sensitization to pain by summarizing the empirical evidence gathered in many separate studies. Moreover, human genetic research on persisting pain emphasizes the immune system as a potential source of important future targets for analgesic drugs directed against persisting pain and demonstrates that contemporary machine‐learned methods offer innovative approaches to knowledge discovery from previous evidence.

## Author contributions

JL, DK and EK conceived and designed the analysis. JL, CL and DK analysed the data. JL, DK, EK and MJP wrote the article. JL, AU, DK and EK involved in discussion of methods and results.
